# Molar Pregnancy Complicated by Impending Thyroid Storm

**DOI:** 10.7759/cureus.19656

**Published:** 2021-11-17

**Authors:** Shorabh Sharma, Sapna Sharma, Liliya Gandrabur, Bushra Amin, Razia Rehmani, Abhilasha Singh

**Affiliations:** 1 Internal Medicine, St. Barnabas Hospital Health System, Bronx, USA; 2 Rheumatology, St. Barnabas Hospital Health System, Bronx, USA; 3 Internal Medicine, Montefiore Medical Center, Wakefield Campus, New York, USA; 4 Radiology, St. Barnabas Hospital Health System, Bronx, USA; 5 Endocrinology, East Valley Diabetes and Endocrinology, San Tan Valley, USA

**Keywords:** beta-human chorionic gonadotropin, pregnancy, hyperthyroidism, pre-eclampsia, hydatidiform mole, thyroid storm

## Abstract

Gestational trophoblastic diseases, which include molar pregnancy, have an increased risk of complications associated with the thyroid gland. This condition is mainly caused by elevated levels of beta-human chorionic gonadotropin produced during pregnancy, which is exaggerated in molar pregnancy and can lead to thyrotoxicosis. Hence, it is important to recognize the signs and symptoms of hyperthyroidism among women of childbearing age to prevent complications such as thyroid storm. Medical management of thyroid storm before surgery is critical to prevent adverse maternal outcomes. Here, we report a rare case of impending thyroid storm induced by molar pregnancy.

## Introduction

Pregnancy is associated with physiological changes in both maternal thyroid gland volume and function because of an interplay among beta-human chorionic gonadotropin (β-hCG), estrogen, and the thyroid gland. Hyperthyroidism occurs in 0.2-0.7% of all pregnancies, with Graves’ disease accounting for 95% of the cases [1]. Hyperthyroidism caused by molar pregnancy is a rare form of pregnancy-induced hyperthyroidism and can lead to a potentially life-threatening complication of thyroid storm. Here, we describe the case of a patient with an impending thyroid storm induced by molar pregnancy.

## Case presentation

**Figure 1:**
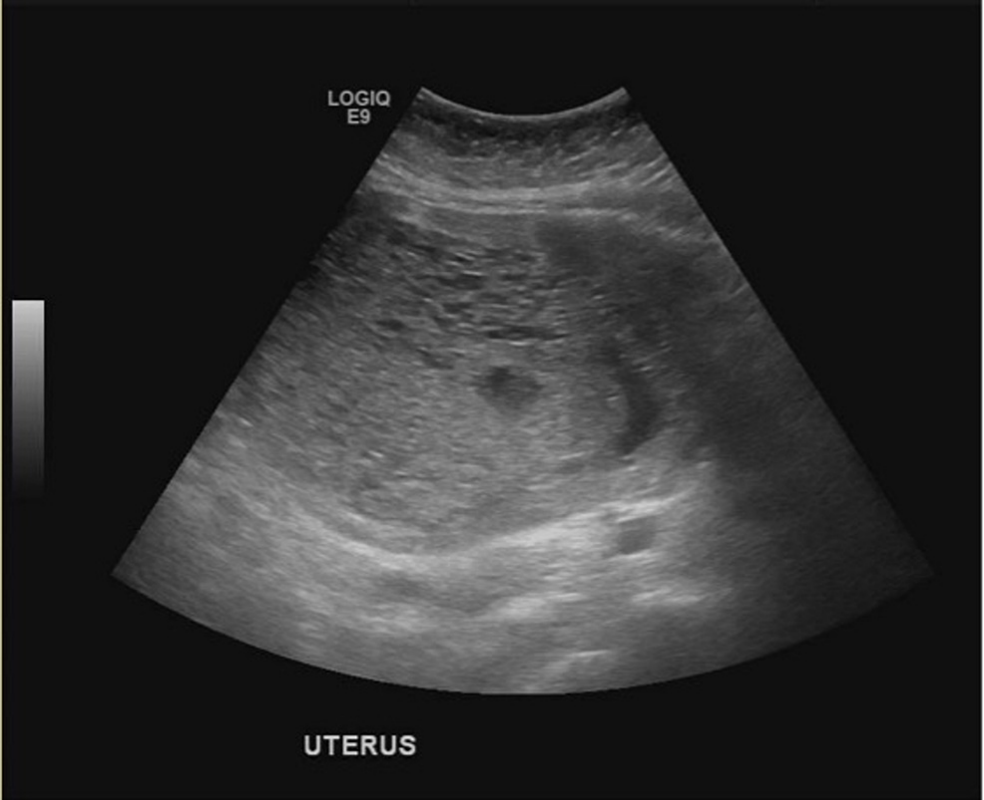
Transabdominal ultrasound showing an enlarged uterus with cystic spaces and absent fetal parts.

A 48-year-old female (gravida 6, para 3023) with a history of pre-eclampsia in a previous pregnancy was referred to the emergency room by her gynecologist at 11 weeks of gestation due to symptoms of headache, nausea, vomiting, vaginal spotting, and elevated blood pressure (BP) concerning for pre-eclampsia. On presentation, she was afebrile with a temperature of 99.6°F, heart rate of 100 beats/minute, and hypertensive with a BP of 167/80 mmHg. Abdominal examination was notable for tenderness to palpation in the right lower quadrant with a palpable uterus of 16-week gestation. Laboratory evaluation revealed β-hCG elevated at 1,771,640 mIU/mL (normal: 0.5-2.9 mIU/mL), elevated transaminases such as aspartate aminotransferase (AST)/alanine aminotransferase (ALT) of 149/178 IU/L (normal: 8-33/4-36 IU/L), as well as thyrotoxicosis with suppressed thyroid-stimulating hormone (TSH) of 0.07 uIU/mL (normal: 0.34-5.60 uIU/mL), elevated free T4 of 4.09 ng/dL (normal: 0.82-1.77 ng/dL), and total T3 of 201 ng/mL (normal: 87-188 ng/mL). Bedside ultrasound demonstrated an enlarged uterus measuring 14.6 × 8.7 × 16.7 cm (normal non-pregnant uterus measures 7.5 × 5 × 2.5 cm), with uterine contents measuring 7.21 × 4.17 cm (Figure 1). CT of the abdomen and pelvis was consistent with the ultrasound findings of enlarged uterus measuring 21.7 cm × 14.6 cm × 11.4 cm, with enhancing nodular and hypodense areas concerning for molar pregnancy. The additional evaluation included CT of the chest and MRI of the brain which did not reveal any foci concerning for metastatic disease.

As the calculated Burch-Wartofsky score of 30 was concerning for impending thyroid storm, she was admitted to the intensive care unit for further management. She was started on intravenous labetalol infusion and magnesium sulfate for blood pressure control and seizure prophylaxis, respectively. Additionally, she received propylthiouracil (PTU) 200 mg every four hours, Lugol’s iodine 80 mg every eight hours, and stress doses of hydrocortisone 100 mg every eight hours, with subsequent improvement in total T3 levels (Table 1). Once BP control was achieved, she was transferred to a tertiary care center where she underwent dilatation and curettage with suction.

**Table 1:** Trend of laboratory values from admission to outpatient follow-up. TSH: thyroid-stimulating hormone; β-hCG: beta-human chorionic gonadotropin; AST: aspartate aminotransferase; ALT: alanine aminotransferase

	Normal range	Day 1	Day 3	Day 6 (Day of the surgery)	Day 9 (Outpatient follow-up)	Day 20 (Outpatient follow-up)
TSH (uIU/mL)	0.34–5.60	0.07	-	-	-	-
Total T3 (ng/mL)	87–188	291	155	133	-	-
Total T4 (ug/L)	6.0–12.0	25.1	24.7	21.7	-	-
Free T4 (ng/dL)	0.82–1.77	4.09	-	-	-	-
Free T4 (pg/mL)	2.0–4.4	9.7	-	-	-	-
β-hCG (mIU/mL)	0.5–2.9	1,771,640	-	757,993	72,831	3,147
AST/ALT (IU/L)	8–33/4–36	149/178	-	131/167	36/55	-

Pathological evaluation of the mass demonstrated an absence of fetal parts with positive p57 staining supporting the diagnosis of a complete hydatidiform mole. She was discharged on methimazole 20 mg twice daily and nifedipine 90 mg daily. On her follow-up visit 10 days later, nifedipine was discontinued and methimazole was reduced to 10 mg daily. She was advised contraception with a plan to initiate methotrexate therapy for chemoprophylaxis.

## Discussion

Gestational trophoblastic diseases (GTDs) are characterized by abnormal trophoblastic proliferation and encompass a spectrum of benign and malignant tumors, including hydatidiform moles (complete or partial), invasive mole, and choriocarcinoma [2]. In the United States, GTDs occur in approximately 121 per 100,000 pregnancies and choriocarcinoma occurs in 3 per 100,000 pregnancies [2]. Patients with GTDs are at risk of developing hyperthyroidism with the subsequent potential complications of pre-eclampsia and thyroid storm [3].

Hydatidiform mole usually presents in the first trimester of pregnancy with signs and symptoms of vaginal bleeding, abdominal cramping, excessive vomiting, fatigue, and a uterus larger than expected for the gestational age. Common risk factors include extremes of maternal age (<15 years and >35 years) and history of hydatidiform mole, viral infections, maternal immune mechanisms, cytogenetic abnormality, nutritional status, multiparity, and oral contraception [4]. Diagnostic findings include elevated β-hCG level and ultrasound of the abdomen and pelvis demonstrating an enlarged uterus with multiple cystic spaces and absent fetal parts. This may resemble a “snowstorm” appearance or a “bunch of grapes” sign [2]. Once the diagnosis of molar pregnancy is confirmed, categorization into partial or complete mole is performed by tissue analysis as different types of hydatidiform moles have a varied risk of developing into choriocarcinoma [5]. Further imaging with CT of the chest, abdomen, and pelvis should be considered to evaluate for metastasis, with the most common sites being lung (80%), vagina (30%), brain (10%), and liver (10%) [6].

In normal pregnancy, elevated circulating levels of hCG in the first trimester can transiently increase T4 secretion, which, in turn, suppresses TSH. The thyrotropic effect of β-hCG is attributed to the structural homology of its β-subunit to TSH. Additionally, hCG and TSH receptors exhibit similar homology of approximately 70% for the transmembrane domain and 45% for the extracellular domains of the receptors. Hyperthyroidism is known to be associated with GTDs because of this cross-reactivity and significantly higher and sustained β-hCG levels compared to normal pregnancy. In addition, β-hCG in molar pregnancy is thought to be less sialylated, leading to a more potent thyrotropic [7-9]. Kaulfers et al. reported that, for every 10,000 mIU/mL increase in serum hCG, TSH decreases by 0.1 mIU/mL and free T4 increases by 0.1 ng/dL [10].

Maternal complications of hyperthyroidism include cardiac failure and thyroid storm. The latter is a rare entity but has a high mortality rate of up to 15-30%, and is associated with fever, tachycardia, agitation, or altered mental status [11]. Burch and Wartofsky developed a scoring system for the early detection of thyroid storm [12]. However, it has limitations because it can be non-specific. It is helpful in categorizing severity and recognizing the early signs of thyroid storm, which are crucial to prevent complications of thyroid storm such as stroke, arrhythmias, rhabdomyolysis, liver dysfunction, and death [13]. Liver dysfunction in thyroid disease can be due to multiple reasons because thyroid hormones are glucuronidated and sulfated in the liver. Excess thyroid hormone can induce hepatocyte apoptosis through a mitochondrial-dependent pathway. The degree of damage in thyroid storm could range from asymptomatic elevation in aminotransferases to acute liver failure [14].

The management of thyroid storm requires an understanding of the steps of thyroid hormone synthesis as well as the drugs that target these steps. PTU or methimazole stops thyroid hormone synthesis by inhibiting thyroid peroxidase and blocks iodine organification, whereas iodine helps to acutely lower thyroid hormone concentration by decreasing the release of the hormone from the gland and inhibiting organification (the Wolff-Chaikoff effect). Iodine is usually used for a short course of approximately 10 days with thionamide administered before initiating therapy. In addition, beta-blockers such as propranolol and glucocorticoids help in decreasing peripheral conversion of T4 to T3 with an additional effect of blunting sympathetic stimulation with propranolol [15]. Definitive management in molar pregnancy is dilatation and curettage for the evacuation of the mole. The pathology is sent for analysis which includes karyotyping. Hysterectomy is the procedure of choice in patients who do not desire further pregnancy. Serial monitoring of quantitative β-hCG is crucial for up to six months for complete moles to evaluate for progression to choriocarcinoma or post-molar gestational trophoblastic neoplasia [16].

## Conclusions

Thyroid imbalances are common in pregnancies, and even more so in molar pregnancy because of sustained increase in β-hCG. Our case highlights the importance of recognizing an impending thyroid storm for prompt management before definitive treatment with dilatation and curettage of the hydatidiform mole can be performed. Because thyroid storm has a high mortality rate of up to 30%, early recognition and comprehensive management of the underlying etiology with a multidisciplinary team of endocrinologists, gynecologists, and critical care physicians are vital for a successful outcome.

## References

[1] (2020). Thyroid disease in pregnancy: ACOG Practice Bulletin, Number 223. Obstet Gynecol.

[2] Altieri A, Franceschi S, Ferlay J, Smith J, La Vecchia C (2003). Epidemiology and aetiology of gestational trophoblastic diseases. Lancet Oncol.

[3] Bruce S, Sorosky J (2020). Gestational trophoblastic disease. https://www.ncbi.nlm.nih.gov/books/NBK470267/.

[4] AlJulaih GH, Muzio MR (2020). Gestational trophoblastic neoplasia. https://www.ncbi.nlm.nih.gov/books/NBK562225/.

[5] Mondal SK, Mandal S, Bhattacharya S, Panda UK, Ray A, Ali SM (2019). Expression of p57 immunomarker in the classification and differential diagnosis of partial and complete hydatidiform moles. J Lab Physicians.

[6] Berkowitz RS, Goldstein DP (1981). Pathogenesis of gestational trophoblastic neoplasms. Pathobiol Annu.

[7] Hershman JM (2004). Physiological and pathological aspects of the effect of human chorionic gonadotropin on the thyroid. Best Pract Res Clin Endocrinol Metab.

[8] Yoshimura M, Hershman JM (1995). Thyrotropic action of human chorionic gonadotropin. Thyroid.

[9] Glinoer D (1997). The regulation of thyroid function in pregnancy: pathways of endocrine adaptation from physiology to pathology. Endocr Rev.

[10] Kaulfers AM, Bhowmick SK (2015). Molar pregnancy causing thyrotoxicosis in a teenage girl with type 1 diabetes mellitus. Glob Pediatr Health.

[11] Moleti M, Di Mauro M, Sturniolo G, Russo M, Vermiglio F (2019). Hyperthyroidism in the pregnant woman: maternal and fetal aspects. J Clin Transl Endocrinol.

[12] Burch HB, Wartofsky L (1993). Life-threatening thyrotoxicosis. Thyroid storm. Endocrinol Metab Clin North Am.

[13] Nussey S, Whitehead S (2001). Endocrinology: an integrated approach. https://pubmed.ncbi.nlm.nih.gov/20821847/.

[14] Khemichian S, Fong TL (2011). Hepatic dysfunction in hyperthyroidism. Gastroenterol Hepatol (N Y).

[15] Cavaliere A, Ermito S, Dinatale A, Pedata R (2009). Management of molar pregnancy. J Prenat Med.

[16] Soper JT (2021). Gestational trophoblastic disease: current evaluation and management. Obstet Gynecol.

